# Artificial intelligence—based diagnosis of oral leukoplakia using deep convolutional neural networks Xception and MobileNet-v2

**DOI:** 10.3389/froh.2025.1414524

**Published:** 2025-03-21

**Authors:** Elakya Ramesh, Anuradha Ganesan, Krithika Chandrasekar Lakshmi, Prabhu Manickam Natarajan

**Affiliations:** ^1^ Department of Oral Medicine and Radiology, SRM Dental College, Chennai, Tamil Nadu, India; ^2^ Department of Clinical Sciences, Center of Medical and Bio-Allied Health Sciences and Research, College of Dentistry, Ajman University, Ajman, United Arab Emirates

**Keywords:** oral premalignant disorder, oral leukoplakia, convolutional neural networks, artificial intelligence, deep learning, diagnostic accuracy

## Abstract

**Objective:**

The present study aims to employ and compare the artificial intelligence (AI) convolutional neural networks (CNN) Xception and MobileNet-v2 for the diagnosis of Oral leukoplakia (OL) and to differentiate its clinical types from other white lesions of the oral cavity.

**Materials and methods:**

Clinical photographs of oral leukoplakia and non-oral leukoplakia lesions were gathered from the SRM Dental College archives. An aggregate of 659 clinical photos, based on convenience sampling were included from the archive in the dataset. Around 202 pictures were of oral leukoplakia while 457 were other white lesions. Lesions considered in the differential diagnosis of oral leukoplakia like frictional keratosis, oral candidiasis, oral lichen planus, lichenoid reactions, mucosal burns, pouch keratosis, and oral carcinoma were included under the other white lesions subset. A total of 261 images constituting the test sample, were arbitrarily selected from the collected dataset, whilst the remaining images served as training and validation datasets. The training dataset were engaged in data augmentation to enhance the quantity and variation. Performance metrics of accuracy, precision, recall, and f1_score were incorporated for the CNN model.

**Results:**

CNN models both Xception and MobileNetV2 were able to diagnose OL and other white lesions using photographs. In terms of F1-score and overall accuracy, the MobilenetV2 model performed noticeably better than the other model.

**Conclusion:**

We demonstrate that CNN models are capable of 89%–92% accuracy and can be best used to discern OL and its clinical types from other white lesions of the oral cavity.

## Introduction

Oral potentially malignant disorders (OPMDs) refers to a class of lesions of the oral mucosa that have an increased risk of developing into cancer. Currently noticed OPMDs that carry a high risk of malignant transformation (MT) include erythroplakia, oral lichen planus (OLP), discoid lupus erythematosus, dyskeratosis congenita, leukoplakia, erythroplakia, actinic cheilitis, palatal lesions of reverse cigar smoking, discoid lupus erythematosus, dyskeratosis congenita, oral lichenoid lesions, and oral graft vs. host disease (1). Shanbhag et al. proposed a new simple definition for leukoplakia as: “a predominantly white, irreversible, non-scrapable lesion of the oral mucosa that cannot be characterized clinically or histopathologically as any other lesion/disease and has increased risk of cancer occurrence than its normal counterpart and is usually associated with consumption of tobacco, betel quid, and alcohol, but otherwise can be of idiopathic in nature” (2).

When multiple white patches or lesions are observed in the oral cavity, accurate diagnosis of leukoplakia is crucial. Based on color and thickness and texture, leukoplakia can be classified into homogeneous and non- homogeneous sub-groups in clinical terms. Uniform white patches with thin, smooth, or wrinkled surfaces that may have tiny fissures or cracks are the hallmark of homogeneous leukoplakia. Literature supports that other clinical facades of homogenous leukoplakia subvariants encompass pumice-stone or velvet-like appearance. Erythroleukoplakia is one of the non-homogenous erythematous type of leukoplakia that is often classified into nodular, granular or speckled variants while verrucous leukoplakia is the other subtype (3).

Histopathological evaluation is vital in identifying the risk of OL transformation to malignancy. Nevertheless, though literature evidences support the use of liquid biopsy, light-based detection systems, salivary biomarker analysis, DNA microarray analysis, spectroscopy techniques and nano diagnostics to assess its chance of progression to cancer, biopsy followed by histologic evaluation is the most reliable (4, 5). Vital staining kits, brush biopsy, salivary diagnostics, optical imaging systems cannot substitute the conventional gold standard technique but can be adjuncts in the diagnosis of leukoplakia (5).

Early detection and treatment assist in the prevention of the disease progression, enhances patients' quality of life, increases survival rates, and lowers morbidity effectively. The advanced stages of oral cancer often involve more invasive treatment which increases morbidity, and cost of treatment and significantly impacts the individual's quality of life. The prognosis of oral cancer worsens in the advanced stages of cancer. The survival rate of early-stage oral cancer is approximately 69.3% but will decrease to 31.2% in the advanced stage. This number has not significantly improved in the past few decades regardless of various treatments. Early detection of oral cancer, is therefore very important as it not only increases the survival rate but also improves the quality of life of patients. The majority of procedures need to be diagnosed by specialists, which is expensive and a barrier to large scale introduction. In this scenario, Artificial intelligence AI-based technologies present chances to drastically lower analysis cost, expedite management, and simultaneously improve diagnostic precision. Finding the best diagnosis at the lowest feasible cost seems to be possible with the combination of web-based interface and explanatory AI techniques (6).

Artificial intelligence is the simulation of human intelligence processes by machines, especially computer systems. John McCarthy, who is regarded as the founder of artificial intelligence, first used the word to refer to machines that were capable of acting in a way that considered to be intelligent without the need for human involvement (7). A subset of artificial intelligence is machine learning (ML) (8). ML uses methods like artificial neural networks (ANN) to anticipate the result based on the dataset that is given to it. These networks, which have linked artificial neurons that receive and process data signals, resemble the structure of the human brain (9). Convolutional neural networks, often known as deep learning networks (DL), are machine learning techniques that use multi-layer neural networks to compute data. Deep learning algorithms can potentially enhance the result by analyzing patterns found in the data (10). Artificial intelligence (AI) has been proposed as a beneficial tool to forecast the disease diagnosis and patient-specific therapy planning.

AI in particular can help dentists make crucial, time-sensitive decisions. It can eliminate the possibility of human mistake in decision-making, resulting in better, more consistent medical care and less stress for dentists (11). The principles of DL involve the standard DL model, convolutional neural network (CNN) using object recognition, and classification of images from the data put in the system. The application of algorithms for picture processing is ubiquitous in medical applications, particularly for the detailed analysis of medical images. These methods first carry out feature extraction of specific images and subsequently conduct target detection or categorize images into established classes to achieve image detection or classification.

It could be challenging for general practitioners to diagnose OL clinically and distinguish it from other white lesions. Additionally, no research has been done to define how well AI does when diagnosing OL using clinical photographs. In order to forecast the lesion's future evolution, we present an AI methodology in our work for the analysis of leukoplakia from standard digital photos and to differentiate its clinical types from other white lesions of the oral cavity.

## Materials and methods

This research was approved by the Ethics Review Board of SRM Dental college, Ramapuram (SRMU/M&HS/SRMDC/2023/PG/014).

### Data preparation

Clinical photographs of oral leukoplakia and non-Oral leukoplakia lesions were gathered from the SRM Dental College and University archive. A total of 659 clinical photos from the archive were included in the dataset, which was created using the convenience sampling method. The pictures were divided into two groups: 202 for lesions related to oral leukoplakia and 457 other white lesions. In this study, histopathological confirmation was obtained for lesions however, clinical diagnosis was primarily based on expert evaluation by experienced oral and maxillofacial physicians. Other white lesions include lesions like frictional keratosis, oral candidiasis, oral lichen planus, lichenoid reactions, mucosal burns, pouch keratosis, scarring and oral carcinoma were considered in the differential diagnosis of oral leukoplakia.

A curated dataset of clinical images of white lesions of the oral cavity were collected. Each image had labels such as Oral leukoplakia—Homogenous, Oral Leukoplakia-Non Homogenous speckled type, Nodular type, verrucous type, other white lesions—frictional keratosis, oral carcinoma, lichen planus, oral candidiasis, etc. A total of 261 photographs were randomly selected for use as the test dataset, while the remaining photographs were used for training and validation datasets.

### Data augmentation

It was performed on the training dataset to enhance the quantity and variation in photographs. Random image augmentation techniques were applied which included reflection, translation, scaling, and flipping. These augmentations expanded the dataset, ensuring the model's exposure to a wide array of potential inputs.

The training process commenced, focusing on the optimization of hyperparameters across epochs. Iteratively, we fine-tuned crucial factors such as learning rates and batch sizes, leveraging the ADAM optimizer. This meticulous calibration was fundamental in crafting a resilient and well-trained model. Each photograph was resized to a fixed dimension of 224 × 224. Finally, the proposed models were trained to build a classification that classifies oral images into three categories.

### CNN for oral leukoplakia and other white lesion diagnosis

CNN is a neural network model commonly used in deep neural networks for visual vision analysis. A fully connected neural network or a recurrent neural network are two more neural network models that excel on many kinds of data.

The widely-used CNN models include AlexNet, VGG-16, Xception, and ResNet-50. For Oral Leukoplakia and other white lesion detection in this investigation, the Xception and MobileNetV2 models were selected due to their low model complexity and high classification accuracy on the ImageNet classification task, which was summarized by Keras. Xception by Google, stands for Extreme version of Inception. With a modified depthwise separable convolution, it is even better than Inception-v3 (also by Google, 1st Runner Up in ILSVRC 2015) for both ImageNet ILSVRC and JFT datasets. The original depthwise separable convolution is the depth wise convolution followed by a pointwise convolution. Depth wise convolution is the channel-wise *n* × n spatial convolution. Pointwise convolution actually is the 1 × 1 convolution to change the dimension. The modified depth wise separable convolution is the pointwise convolution followed by a depth wise convolution. This modification was motivated by the inception module in Inception-v3 that 1 × 1 convolution is done first before any *n* × *n* spatial convolutions. Thus, it is a bit different from the original one. (*n* = 3 here since 3 × 3 spatial convolutions are used in Inception-v3). The modified depth wise separable convolution with different activation units is tested. The Xception without any intermediate activation has the highest accuracy.

In MobileNetV2, there are two types of blocks. One is residual block with stride of another one is block with stride of 2 for downsizing. There are 3 layers for both types of blocks. This time, the first layer is 1 × 1 convolution with ReLU6. The second layer is the depth wise convolution. The third layer is another 1 × 1 convolution but without any non-linearity. It is claimed that if ReLU is used again, the deep networks only have the power of a linear classifier on the non-zero volume part of the output domain. And there is an expansion factor *t*. And *t* = 6 for all main experiments. If the input got 64 channels, the internal output would get 64 × *t* = 64 × 6 = 384 channels.

The model was trained to classify three classes of data (homogenous leukoplakia, non-homogenous leukoplakia and other white lesions), as previously mentioned. For training process Epochs—50, Batch size—64, Optimizer—Adam, Input Image Size—224 × 224 were used in Xception model and Epochs—50, Batch size—32, Optimizer—Adam, Input Image Size—224 × 224 were used in MobileNetV2 model. Throughout the training process, the inference loss and accuracy from the validation dataset were used to assess the model's performance at every epoch. The testing dataset's photographs were diagnosed using the weights of the epoch with the lowest validation loss after the training completed.

**Figure 1:**
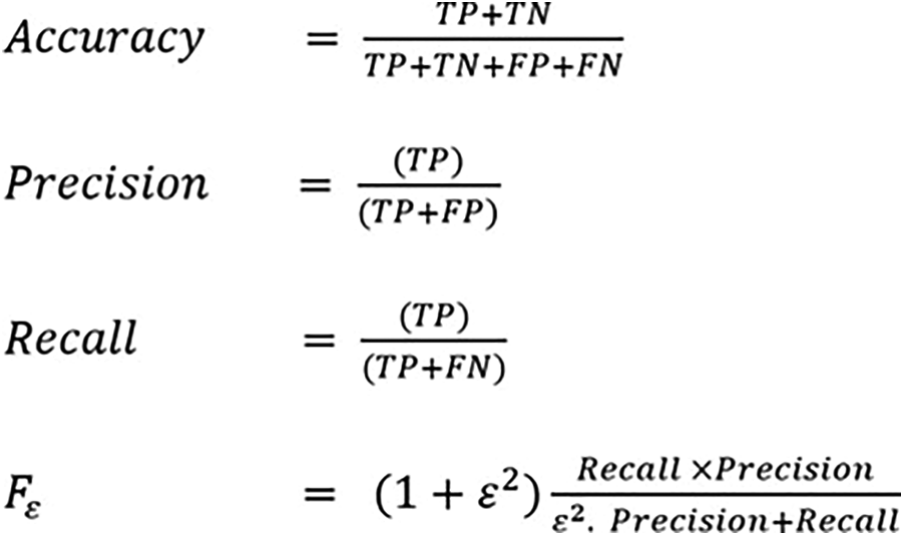
Metrics of performance for CNN models in homogenous leukoplakia, non homogenous leukoplakia and other white lesions diagnosis. CNN, convolutional neural network; FN, false negative; FP, false positive; TN, true negative; TP, true positive.

Performance metrics for the CNN model performance included accuracy, precision, recall, and f1_score. Accuracy measures the analysis of TP and TN to the total no. of test images. Precision is the estimation analysis of true positive to the aggregate value of true positive and false positive rate. Recall is the estimation analysis of true positive rate to the aggregate value of the true positive and false negative rate. F-Score: F-Measure is the harmonic mean of recall and precision. The equation for each metric is summarized in (Figure 1).

## Results

**Figure 2:**
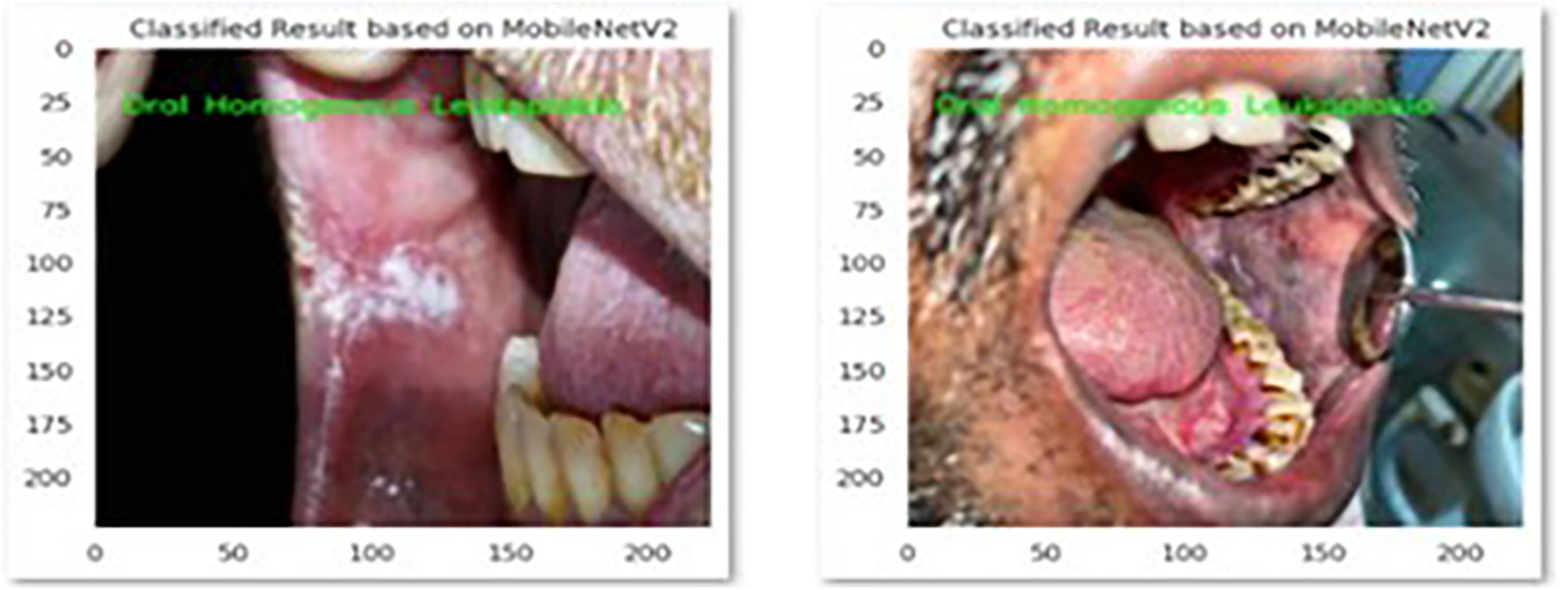
This picture presents MobileNetV2 model's correct identification of oral homogenous leukoplakia.

**Figure 3:**
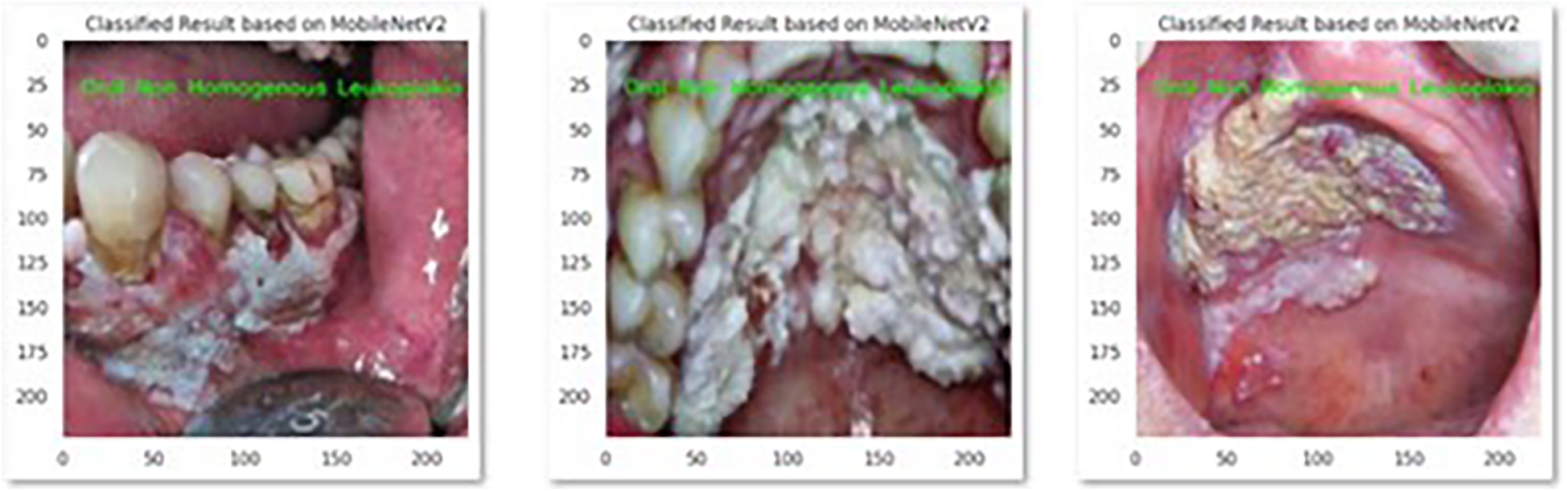
This picture presents MobileNetV2 model's correct identification of oral non homogenous leukoplakia.

The performance of the CNN model MobileNetV2 and Xception in the correct identification and its classification into three groups i.e., Homogenous leukoplakia, non-homogenous leukoplakia and other white lesions was demonstrated in Figure 2 and Figure 3.

The performances of the two CNNs for homogenous OL, non-Homogenous OL and other white lesions diagnosis on the test dataset after training using the same training parameters are summarized in (Table 1).

**Table 1:** Confusion matrix of each model in predicting homogenous OL, non-homogenous OL and other white lesions using the photographs in the test dataset.

Model		Actual homogenous OL	Actual non homogenous OL	Actual other oral white lesions
Xception	Predicted homogenous OL	83	4	3
	Predicted non homogenous OL	3	65	5
	Predicted other white lesions	6	8	84
MobileNetV2	Predicted homogenous OL	83	3	2
	Predicted non homogenous OL	7	71	5
	Predicted other white lesions	2	3	85

OL, oral leukoplakia.

**Figure 4:**
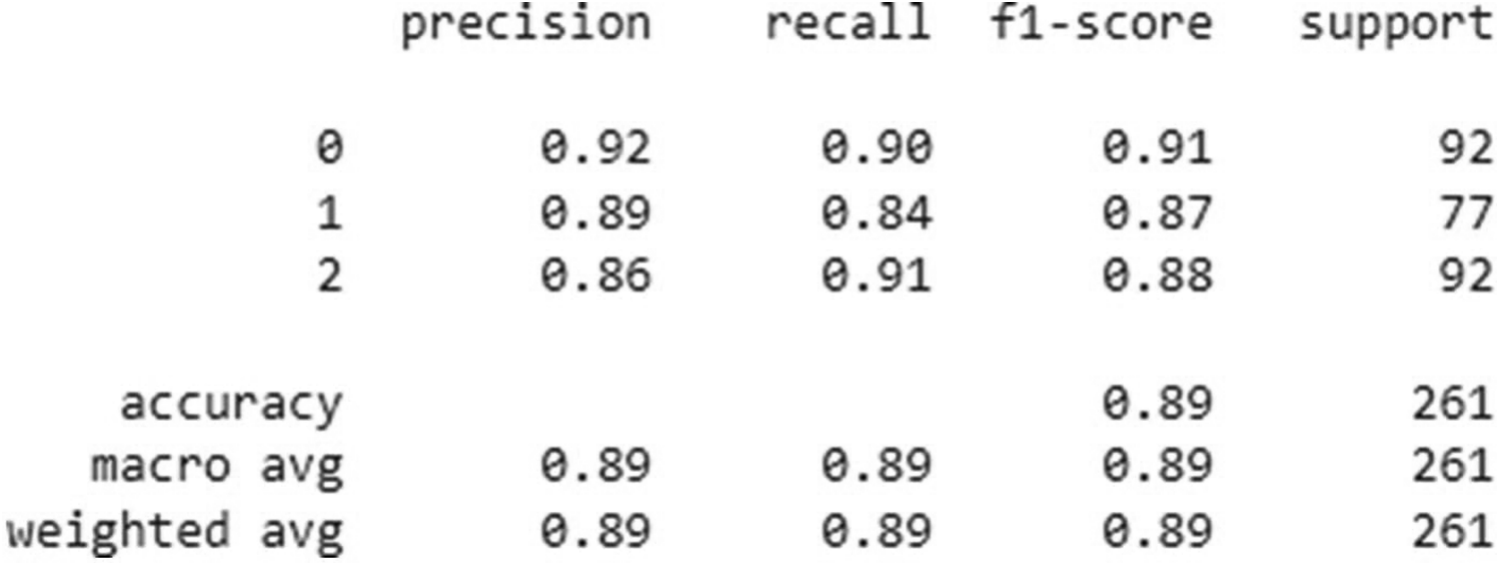
Classification performance metrics of the Xception model for three groups- homogenous leukoplakia, non-homogenous leukoplakia and other white lesions (0, 1, and 2). The table presents precision, recall, and F1-score for each group, along with the number of true instances (support). The model achieved an overall accuracy of 89%, with a macro and weighted average F1-score of 0.89, indicating balanced performance across all classes.

**Figure 5:**
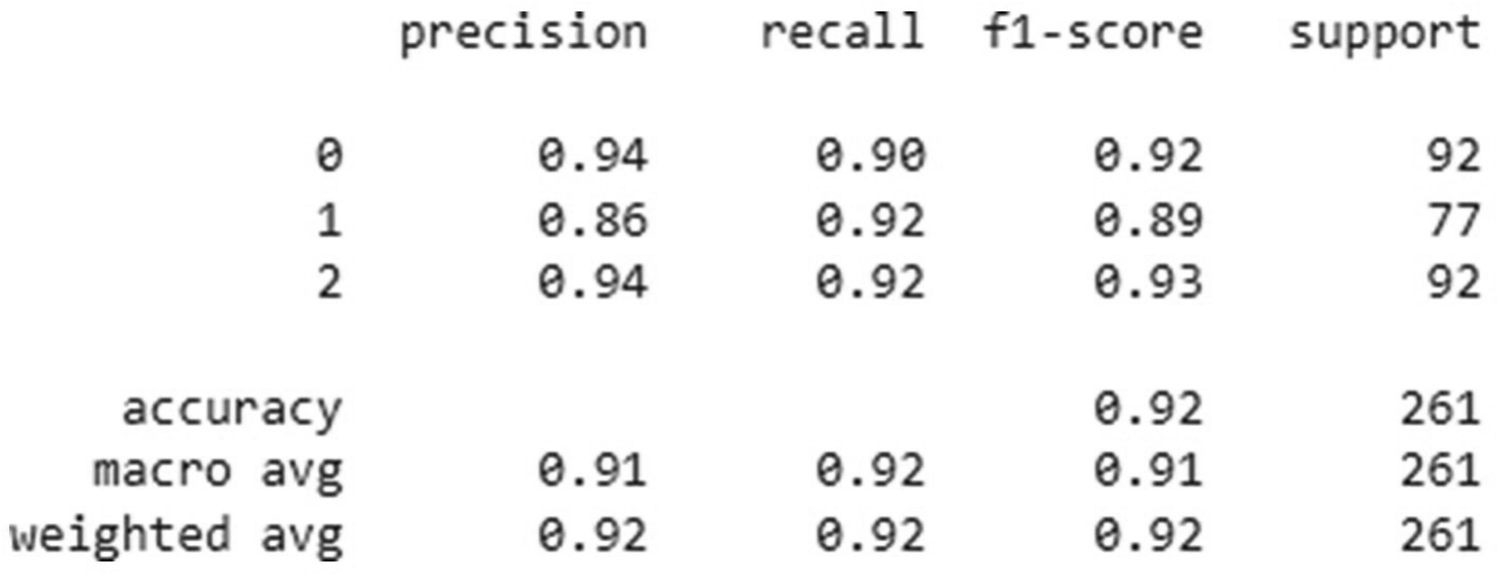
Classification performance metrics of the MobNetV2 model for three groups—homogenous leukoplakia, non-homogenous leukoplakia and other white lesions (0, 1, and 2). The table presents precision, recall, and F1-score for each group, along with the number of true instances (support). The model achieved an overall accuracy of 92%, with macro and weighted average F1-scores of 0.91 and 0.92, respectively, indicating improved classification performance compared to previous results. Notably, group 1 showed higher recall (0.92), suggesting better sensitivity in detecting this category.

The overall accuracy, precision, recall and F1-score of two CNN model's was given in Figures 4, 5.

MobileNetV2 outperformed Xception in sensitivity, particularly for non-homogeneous OL (92%) and other white lesions (94%), indicating its superior ability to correctly identify these cases. In contrast, Xception demonstrated slightly lower sensitivity for non-homogeneous OL (85%) but remained strong in detecting homogeneous OL (89%) and other white lesions (91%).

Both models exhibited high specificity across all categories, with Xception achieving 96% for homogeneous OL and 94% for non-homogeneous OL, while MobileNetV2 showed 96% for homogeneous OL and 93% for non-homogeneous OL. Notably, MobileNetV2 had the highest specificity (95%) for other white lesions, making it slightly more reliable in distinguishing them from OL.

Overall, MobileNetV2 demonstrated a more balanced performance with higher sensitivity and specificity, making it a preferable model for diagnosing OL and differentiating it from other white lesions as shown in Table 2.

**Table 2:** Mobilenetv2 showed higher sensitivity for non-homogeneous OL and other white lesions, making it better at correctly detecting these cases.

Model	Homogeneous OL sensitivity	Non-homogeneous OL sensitivity	Other white lesions sensitivity	Homogeneous OL specificity	Non-homogeneous OL specificity	Other white lesions specificity
Xception	89%	85%	91%	96%	94%	92%
MobileNetV2	90%	92%	94%	96%	93%	95%

Xception had slightly lower sensitivity for non-homogeneous OL but was still strong in homogeneous OL detection. Specificity was high for both models, with MobileNetV2 showing slightly better specificity for other white lesions detection.

The majority of the OL and other white lesions photographs had accurate diagnoses, as seen in Figures 1, 2. In this present study classifying images of homogeneous leukoplakia, non-homogenous leukoplakia, and other white lesions was performed better with the Xception and MobileNetV2 models. The performance of the MobileNetV2 model was significantly higher than that of the other models in terms of overall accuracy and F1-score of 89% and 92%, respectively. For instance, the overall accuracy of the MobileNetV2 model was up to 6% higher than that of the other models. The overall model performance can still be considered satisfactory with such a limited dataset.

## Discussion

Oral leukoplakia, with a prevalence rate of 2%- 3% worldwide, is the most common potentially malignant lesion of the oral cavity. Patients older than 40 years of age are more likely to have it, and malignant transformation rate have been found to range from 1% to 40%, with an average of 13% (12, 13). Histological examination is the gold standard for diagnosing oral leukoplakia and should always be performed along with a clinical examination of the lesions, particularly if the patient has high-grade leukoplakia (14). Molecular testing of saliva or tissue would be excellent adjuncts to reliably identify leucoplakias that are either at higher risk or lower risk to develop into cancer.

Our study utilized a dataset of clinical photographs grouped as training, validation, and testing sets. Data augmentation techniques could expand the data volume and get other potential inputs. Xception and MobileNet-v2 CNN models were trained and evaluated using metrics such as accuracy, precision, recall, and F1-score. Tanriver et al. developed a two-stage algorithm using deep learning to identify and categorize oral lesions into three groups (benign, OPMD and malignancy). Their initial findings show that a deep learning- based method for the automatic real- time identifications and categorization of oral lesions is feasible (15).

Clinical diagnosis performed by human experts was thought to be the gold standard until AI systems that used CNN were developed and showed better outcomes (16). Many sectors have examined AI strategies using CNN for diagnosing diseases (classification) using radiographs, clinical examination, or histopathology (17). Previous research has looked into the use of CNN for skin lesion diagnosis based on clinical appearance and color (18, 19). Diagnostic guidelines for lesions of the skin and oral mucosa are comparable. The criteria for diagnosing oral lesions include variations in color, such as white, red, white- red, brown-black, and yellow, as well as variations in texture, such as ulcerated and vesiculobullous (20).

Oral leukoplakia is generally asymptomatic, presenting as a painless white patch that cannot be scraped off. However, non-homogeneous leukoplakia may have irregular borders, verrucous or ulcerated areas, and can cause mild discomfort, especially with secondary infection or trauma. It is also more clinically aggressive with a higher risk of malignant transformation than homogeneous leukoplakia. In older women, it is more prevalent and has the greatest potential to develop into cancer (21). The application of CNN in OL diagnosis has not been published previously. Our study was the first to apply CNN methods for the diagnosis of Oral Leukoplakia and to differentiate its clinical types from other white lesions of the oral cavity using photographs in a classification model.

Tobacco usage is the main exogenous cause of oxidative stress associated with oral pre-cancerous and malignant conditions. The mucosal surface is predisposed to malignant transformation by prolonged exposure to the carcinogens nitrosamine and benzopyrene found in tobacco and areca nut (22). The overall malignant transformation rate of oral is 10.9% (23). Consequently, early detection of oral leukoplakia is crucial for both prevention of oral cancer and timely treatment. Surgical excision or laser surgery should be the first choice of treatment for lesions exhibiting moderate to severe dysplasia, particularly if the lesion is located on the ventral and lateral borders of the tongue, soft palate, floor of the mouth, or oropharynx. Lesions on various anatomic areas should require careful surveillance and follow-up. For proliferative verrucous leukoplakia and erythroleukoplakia, surgical excision is the recommended course of treatment (24). A delayed diagnosis of OL leads to a large untreated lesion that lowers quality of life. The varied range of clinical presentations of oral leukoplakia makes clinical diagnosis challenging for general practitioners who are not experienced in evaluating soft tissue lesions. The differential diagnosis of oral leukoplakia includes candidiasis, chemical burn, leukoedema, lichen planus, lupus erythematosus, frictional keratosis and oral cancer (25).

Histopathological examination and immunofluorescence tests can provide a conclusive diagnosis of OL. In order to improve the specificity and sensitivity of diagnostic techniques for detecting white, red, and ulcerated lesions during the diagnosis of oral cancer or OPMD, autofluorescence and chemiluminescence have been utilized. These methods produce inconsistent outcomes and are subjective as it depends on the individual's equipment literacy (26). The CNN models will be useful in the advancement of telemedicine and tele-dentistry. The systematic review by Mohammad et al. evaluated the performance of artificial intelligence (AI) models in detecting dental caries on oral photographs and inferred that artificial intelligence (AI) for the automatic detection of dental caries may enable tele dentistry and patient-clinician contact while objectively validating diagnosis (27). A study by Wilsonong et al. demonstrated the promising potential of using CT to opportunistically predict and classify osteoporosis without the need for DEXA using artificial intelligence (28).

This study has implemented artificial intelligence in the diagnosis of oral leukoplakia and to differentiate its clinical types.

A systematic review by Marta Mazur et al. concluded that diagnostic techniques based on medical image analysis—such as autofluorescence, optical imaging, chemiluminescence, and vital stain colorants—cannot replace biopsy, which remains the gold standard for diagnosing OPMDs and OSCC. However, in our study, AI-based deep learning demonstrated higher diagnostic accuracy for oral premalignant lesion, suggesting its potential as an early, noninvasive screening tool while complementing, rather than replacing, the biopsy technique (29). The variety in lesion appearance, as well as subjectivity in diagnosis, make accurate diagnosis even more difficult (30). The novelty of this study lies in its focus on differentiating oral leukoplakia (OL) and its clinical types from other white oral lesions using deep convolutional neural networks (CNNs), specifically Xception and MobileNetV2. While Achararit et al. (2023) demonstrated AI-based diagnosis for oral lichen planus, our study expands the application of CNNs by addressing the broader challenge of distinguishing OL from multiple clinically similar other white lesions. Additionally, this study provides a comparative analysis of CNN architectures, revealing that MobileNetV2 outperforms Xception in diagnostic accuracy (89%–92%) and F1-score. This advancement underscores the potential of AI-driven diagnostic tools in improving clinical decision-making for oral potentially malignant disorders (31).

Using a smartphone to apply the CNN technique could be useful for tele dentistry or for screening lesions prior to consultation or expert referral. In the future, a thorough clinical examination and history-taking combined with the usage of AI technology and CNN as diagnostic aids may be utilized to diagnose diseases. In this work, we created a novel dataset that can be utilized to train machine learning models for the diagnosis of OL and other white lesions from clinical photographs. MobileNetV2 and Xception are deep learning models available in TensorFlow/Keras, which are open-source. However, the cost depends on whether training/inference is performed on local hardware or cloud-based services like Google Cloud. When Google Cloud Platform (GCP) was used for training or inference, the cost varied based on compute engine, google cloud storage, AI platform training and inference cost. According to the demonstration, CNN models may attain an accuracy of 89%–92% on a small dataset. Among the two models, the MobileNetV2 model performed the best in terms of both accuracy and F1-score. There were some limitations on this study due to the limited data set, bias due to convenience sampling and time constraint. Differences in lighting, angle, image resolution, and quality could have influenced model performance. The study used an internal dataset from a single institution, limiting the model's applicability to external populations. Testing on multi-center datasets would improve robustness. Our study demonstrates novelty by applying advanced deep learning models (Xception and MobileNet-v2) to accurately differentiate oral leukoplakia from other white lesions using clinical photographs. This addresses a crucial clinical need, leveraging cutting-edge AI techniques for improved diagnostic accuracy and potentially leading to earlier detection and better patient outcomes.

## Conclusion

In conclusion, although the use of CNN models such as MobileNetV2 shows great promise in diagnosing oral potentially malignant disorders, further research is required to address the current limitations, validate the models across diverse settings, and explore their integration into clinical practice. Amidist the continued advancements in AI and imaging technologies, these models could become a valuable tool in early diagnosis and management, improving patient outcomes and reducing the burden on healthcare professionals. Future studies should also explore the integration of multimodal approaches combining imaging with clinical and histopathological data to further refine AI-assisted diagnostics.

## Data Availability

The raw data supporting the conclusions of this article will be made available by the authors, without undue reservation.

## References

[1] Kumari P Debta P Dixit A. Oral potentially malignant disorders: etiology, pathogenesis, and transformation into oral cancer. Front Pharmacol. (2022) 13:825266. 10.3389/fphar.2022.825266 35517828 PMC9065478

[2] Shanbhag VK. New definition proposed for oral leukoplakia. Dent Res J (Isfahan). (2017) 14(4):297. 10.4103/1735-3327.211627 28928786 PMC5553260

[3] Van der Waal I. Oral leukoplakia; a proposal for simplification and consistency of the clinical classification and terminology. Med Oral Patol Oral Cir Bucal. (2019) 24(6):e799. 10.4317/medoral.23372 31655843 PMC6901141

[4] Rubert A Bagán L Bagán JV. Oral leukoplakia, a clinical-histopathological study in 412 patients. J Clin Exp Dent. (2020) 12(6):e540. 10.4317/jced.57091 32665812 PMC7335600

[5] Mohammed F Fairozekhan AT. Oral leukoplakia. In: Fairozekhan AT, editor. StatPearls. Treasure Island, FL: StatPearls Publishing (2024). Available at: https://www.statpearls.com/ArticleLibrary/viewarticle/12345 (Accessed March 11, 2025). 28723042

[6] Singh A Sengupta S Lakshminarayanan V. Explainable deep learning models in medical image analysis. J Imaging. (2020) 6(6):52. 10.3390/jimaging6060052 34460598 PMC8321083

[7] Nilsson NJ. The Quest for Artificial Intelligence. Cambridge, UK: Cambridge University Press (2009).

[8] Khanagar SB Al-Ehaideb A Maganur PC Vishwanathaiah S Patil S Baeshen HA Developments, application, and performance of artificial intelligence in dentistry–a systematic review. J Dent Sci. (2021) 16(1):508–22. 10.1016/j.jds.2020.06.019 33384840 PMC7770297

[9] Park WJ Park JB. History and application of artificial neural networks in dentistry. Eur J Dent. (2018) 12(4):594–601. 10.4103/ejd.ejd_325_18 30369809 PMC6178664

[10] Sarker IH. Deep learning: a comprehensive overview on techniques, taxonomy, applications and research directions. SN Comput Sci. (2021) 2(6):420. 10.1007/s42979-021-00815-1 34426802 PMC8372231

[11] Shan T Tay FR Gu L. Application of artificial intelligence in dentistry. J Dent Res. (2021) 100(3):232–44. 10.1177/0022034520969115 33118431

[12] Warnakulasuriya S Kujan O Aguirre-Urizar JM Bagan JV González-Moles MÁ Kerr AR Oral potentially malignant disorders: a consensus report from an international seminar on nomenclature and classification. Oral Dis. (2021) 27(8):1862–80. 10.1111/odi.13704 33128420

[13] González-Arriagada WA Canedo-Marroquin G Adorno-Farías D Fernández-Ramires R. New insights into the role of the oral leukoplakia microenvironment in malignant transformation. Front Oral Health. (2024) 5:1363052. 10.3389/froh.2024.1363052 38450102 PMC10914962

[14] Odell E Kujan O Warnakulasuriya S Sloan P. Oral epithelial dysplasia: recognition, grading and clinical significance. Oral Dis. (2021) 27(8):1947–76. 10.1111/odi.13993 34418233

[15] Tanriver G Soluk Tekkesin M Ergen O. Automated detection and classification of oral lesions using deep learning to detect oral potentially malignant disorders. Cancers (Basel). (2021) 13(11):2766. 10.3390/cancers13112766 34199471 PMC8199603

[16] Esteva A Kuprel B Novoa RA Ko J Swetter SM Blau HM Dermatologist-level classification of skin cancer with deep neural networks. Nature. (2017) 542(7639):115–8. 10.1038/nature21056 28117445 PMC8382232

[17] Alabi RO Bello IO Youssef O Elmusrati M Mäkitie AA Almangush A. Utilizing deep machine learning for prognostication of oral squamous cell carcinoma-a systematic review. Front Oral Health. (2021) 2:686863. 10.3389/froh.2021.686863 35048032 PMC8757862

[18] Lyakhov PA Lyakhova UA Nagornov NN. System for the recognizing of pigmented skin lesions with fusion and analysis of heterogeneous data based on a multimodal neural network. Cancers (Basel). (2022) 14(7):1819. 10.3390/cancers14071819 35406591 PMC8997449

[19] Ramadan R Aly S Abdel-Atty M. Color-invariant skin lesion semantic segmentation based on modified U-net deep convolutional neural network. Health Inf Sci Syst. (2022) 10(01):17. 10.1007/s13755-022-00185-9 35978865 PMC9376187

[20] Warnakulasuriya S. Oral potentially malignant disorders: a comprehensive review on clinical aspects and management. Oral Oncol. (2020) 102:104550. 10.1016/j.oraloncology.2019.104550 31981993

[21] Holmstrup P Vedtofte P Reibel J Stoltze K. Oral premalignant lesions: is a biopsy reliable? J Oral Pathol Med. (2007) 36(5):262–6. 10.1111/j.1600-0714.2007.00513.x 17448135

[22] Ganesan A Kumar G. Assessment of lipid peroxides in multiple biofluids of leukoplakia and oral squamous cell carcinoma patients-a clinico-biochemical study. J Clin Diagn Res. (2014) 8(8):ZC55. 10.7860/JCDR/2014/10200.4768 25302269 PMC4190795

[23] Ramaswamy P Kiran CS Raju BM Kiranmai MS. Malignant transformation rate of oral leukoplakia: a meta analysis. J Indian Acad Oral Med Radiol. (2020) 32(4):399–404. 10.4103/jiaomr.jiaomr_63_20

[24] Nadeau C Kerr AR. Evaluation and management of oral potentially malignant disorders. Dent Clin North Am. (2018) 62(1):1–27. 10.1016/j.cden.2017.08.001 29126487

[25] Huber MA. Adjunctive diagnostic techniques for oral and oropharyngeal cancer discovery. Dent Clin North Am. (2018) 62(1):59–75. 10.1016/j.cden.2017.08.004 29126494

[26] Van der Waal I. Oral potentially malignant disorders: is malignant transformation predictable and preventable? Med Oral Patol Oral Cir Bucal. (2014) 19(4):e386. 10.4317/medoral.20205 24905952 PMC4119315

[27] Mohammad-Rahimi H Motamedian SR Rohban MH Krois J Uribe SE Mahmoudinia E Deep learning for caries detection: a systematic review. J Dent. (2022) 122:104115. 10.1016/j.jdent.2022.104115 35367318

[28] Ong W Liu RW Makmur A Low XZ Sng WJ Tan JH Artificial intelligence applications for osteoporosis classification using computed tomography. Bioengineering. (2023) 10(12):1364. 10.3390/bioengineering10121364 38135954 PMC10741220

[29] Mazur M Ndokaj A Venugopal DC Roberto M Albu C Jedliński M *In vivo* imaging-based techniques for early diagnosis of oral potentially malignant disorders—systematic review and meta-analysis. Int J Environ Res Public Health. (2021) 18(22):11775. 10.3390/ijerph182211775 34831531 PMC8622517

[30] Khong B Ferlito S Quek S Conte G Ingrassia A Lechien JR Past, present, and future diagnostic methods for the early noninvasive detection of oral premalignant lesions: a state of the art and systematic review. Ear Nose Throat J. (2024):01455613241245204. 10.1177/01455613241245204 38695398

[31] Achararit P Manaspon C Jongwannasiri C Phattarataratip E Osathanon T Sappayatosok K. Artificial intelligence-based diagnosis of oral lichen planus using deep convolutional neural networks. Eur J Dent. (2023) 17(4):1275–82. 10.1055/s-0042-1760300 36669652 PMC10756816

